# Tanshinone I Inhibits Oxidative Stress–Induced Cardiomyocyte Injury by Modulating Nrf2 Signaling

**DOI:** 10.3389/fphar.2021.644116

**Published:** 2021-05-18

**Authors:** Yu-Ting Wu, Ling-Peng Xie, Yue Hua, Hong-Lin Xu, Guang-Hong Chen, Xin Han, Zhang-Bin Tan, Hui-Jie Fan, Hong-Mei Chen, Jun Li, Bin Liu, Ying-Chun Zhou

**Affiliations:** ^1^ School of Traditional Chinese Medicine, Southern Medical University, Guangzhou, China; ^2^ Department of Traditional Chinese Medicine, Binzhou Medical University Hospital, Binzhou, China; ^3^ Department of Traditional Chinese Medicine, Nanfang Hospital, Southern Medical University, Guangzhou, China; ^4^ Department of Traditional Chinese Medicine, Nanfang Hospital (Zengcheng Branch), Southern Medical University, Guangzhou, China; ^5^ Guangzhou Institute of Cardiovascular Disease, The Second Affiliated Hospital of Guangzhou Medical University, Guangzhou, China; ^6^ TCM Health Construction Department of Yangjiang People’s Hospital, Yangjiang, China

**Keywords:** Tanshinone I, oxidative stress, cardiomyocytes, apoptosis, Nrf2

## Abstract

Cardiovascular disease, a disease caused by many pathogenic factors, is one of the most common causes of death worldwide, and oxidative stress plays a major role in its pathophysiology. Tanshinone I (Tan I), a natural compound with cardiovascular protective effects, is one of the main active compounds extracted from *Salvia miltiorrhiza*. Here, we investigated whether Tan I could attenuate oxidative stress and oxidative stress–induced cardiomyocyte apoptosis through Nrf2/MAPK signaling *in vivo* and *in vitro*. We found that Tan I treatment protected cardiomyocytes against oxidative stress and oxidative stress–induced apoptosis, based on the detection of relevant oxidation indexes such as reactive oxygen species, superoxide dismutase, malondialdehyde, and apoptosis, including cell viability and apoptosis-related protein expression. We further examined the mechanisms underlying these effects, determining that Tan I activated nuclear factor erythroid 2 (NFE2)–related factor 2 (Nrf2) transcription into the nucleus and dose-dependently promoted the expression of Nrf2, while inhibiting MAPK signaling activation, including P38 MAPK, SAPK/JNK, and ERK1/2. Nrf2 inhibitors in H9C2 cells and Nrf2 knockout mice demonstrated aggravated oxidative stress and oxidative stress–induced cardiomyocyte injury; Tan I treatment suppressed these effects in H9C2 cells; however, its protective effect was inhibited in Nrf2 knockout mice. Additionally, the analysis of surface plasmon resonance demonstrated that Tan I could directly target Nrf2 and act as a potential Nrf2 agonist. Collectively, these data strongly indicated that Tan I might inhibit oxidative stress and oxidative stress–induced cardiomyocyte injury through modulation of Nrf2 signaling, thus supporting the potential therapeutic application of Tan I for oxidative stress–induced CVDs.

## Introduction

Cardiovascular disease (CVD), a disease caused by many pathogenic factors, is one of the most common causes of death worldwide (Bansilal et al., 2015). Various CVDs have been associated, at least partially, with increased oxidative stress, which plays a major role in the pathophysiology of cardiac disorders (Senoner and Dichtl, 2019). Several antioxidants such as coenzyme Q10 (CoQ10), vitamin C, and vitamin E have shown preventive and therapeutic benefits against different forms of CVD caused by reactive oxygen species (ROS) generated during excessive oxidative stress. However, poor biopharmaceutical properties and variable pharmacokinetics of several antioxidants have limited their use as therapeutic agents (Jain et al., 2015). Therefore, the exploration of effective antioxidants and their underlying mechanisms may also provide an opportunity for CVD treatment.

*Salvia miltiorrhiza* is widely used clinically to treat CVDs in China to promote blood circulation and reduce blood stasis (Wang et al., 2017). Tanshinone I (Tan I), one of the active components of *Salvia miltiorrhiza*, has a variety of pharmacological effects for preventing and treating CVDs, including antioxidative and antiapoptotic effects (Dai et al., 2017; Guo and Li, 2018). However, the mechanisms underlying its protection against oxidative stress–induced myocardial injury have not been fully elucidated. Combined with the pharmacological effects of Tan I, an in-depth study of the mechanism of Tan I against oxidative stress and oxidative stress–induced cardiomyocyte injury has potential therapeutic significance for its use in the treatment of CVDs.

Nuclear factor erythroid 2 (NFE2)–related factor 2 (Nrf2), a major regulator of oxidation resistance, plays an important role in coordinating the antioxidant response and maintaining redox homeostasis (Ma, 2013). Numerous studies with *in vitro* and *in vivo* CVD models have proven that activation of Nrf2 is critical in the suppression of oxidative stress, including oxidative stress–induced cardiomyocyte apoptosis, suggesting that Nrf2 plays a pivotal role in protection against CVDs (Reuland et al., 2013; Barančík et al., 2016). Nrf2 knockout (Nrf2^−/−^) cardiomyocytes were significantly more vulnerable to oxidative stress–induced cell injury than wild-type (Nrf2^+/+^) cardiomyocytes, and treatment of Nrf2^+/+^ cardiomyocytes with synthetic Nrf2 activator 3H-1, 2-dithiole-3-thione (D3T) upregulated cellular defenses and protected cells against oxidative stress–induced death (Peng and Li, 2002; Cao et al., 2006), which was not observed when Nrf2^−/−^ cardiomyocytes were treated with D3T. These results strongly suggested that Nrf2 activators may have significant therapeutic potential against CVDs. Additionally, increasing evidence has indicated that ROS could mediate activation of MAPK signaling, including P38 MAPK, ERK1/2, and SAPK/JNK cascades to promote cardiomyocyte apoptosis and also exert pivotal functions in the myocardial injury process (Guo et al., 2018; Wen et al., 2018).

However, whether Tan I inhibits oxidative stress–induced cardiomyocyte apoptosis through modulation of Nrf2/MAPK signaling remains to be determined. Tert-butyl hydroperoxide (TBHP), the most common oxidant used to construct cell oxidative stress injury models *in vitro*, induces cardiomyocyte apoptosis (Bi et al., 2018; Fan et al., 2020). In addition, previous studies have indicated that isoproterenol (ISO), a beta-adrenoceptor agonist, has the effect of leading to oxidative stress–induced myocardial injury by causing imbalance between the antioxidants and oxidants in the myocardium (Song et al., 2020; Zhang et al., 2020). Therefore, in this study, TBHP-induced H9C2 cell apoptosis and ISO-induced myocardial injury in mice were used to construct *in vitro* and *in vivo* models, respectively, which were then used to investigate the potential involvement of Nrf2/MAPK signaling in the protective effects of Tan I against oxidative stress and oxidative stress–induced cardiomyocyte apoptosis.

## Materials and Methods

### Materials

Nrf2 (16396-1-AP) antibody was purchased from Proteintech (Chicago, IL, United States). p-ERK1/2 (#4370), p-SAPK/JNK (#4668), p-P38 MAPK (#4511), cleaved caspase3 (#9664), caspase3 (#9662), and GAPDH (#5174) antibodies were purchased from Cell Signaling Technology (Beverly, MA, United States). BAX (AF0120), Bcl-2 (AF6139), and p-ROS (AF8452) antibodies were purchased from Affinity Bioscience (Cincinnati, OH, United States). Tan I was purchased from Chengdu Mansite Biotechnology (Chengdu, China). The H9C2 cell line was purchased from the Institute of Biochemistry and Cell Biology Cell Bank (Shanghai Institutes for Biological Sciences, Chinese Academy of Sciences, Shanghai, China). ML385, PD98059, SB203580, and SP600125 were purchased from MedChemExpress (Monmouth Junction, NJ, United States). The CellTiter 96^®^ Aqueous One Solution Cell Proliferation Assay (MTS) was purchased from Promega (Madison, WI, United States). The Reactive Oxygen Species Assay Kit and Total Superoxide Dismutase Assay Kit were purchased from Beyotime Biotechnology (Shanghai, China). The Malondialdehyde (MDA) Assay Kit was purchased from Nanjing Jincheng Bioengineering Institute (Nanjing, China). The Annexin V-FITC Apoptosis Assay Kit was purchased from AbsinBioscienceInc (Shanghai, China). The TUNEL Apoptosis Assay Kit was purchased from BD Biosciences (Franklin Lakes, NJ, United States).

### Surface Plasmon Resonance Analysis

To detect the interaction between Tan I and Nrf2, a PlexArray HT A100 SPR instrument (Plexera LLC, Woodinville, WA, United States) was used to monitor the process in real time. In brief, a 3D dextran chip with immobilized Nrf2 was inserted into the flow chamber, and different concentrations of Tan I were evaluated. Phosphate-buffered saline (PBS, pH =7.4) was used as the running buffer, while 10 mM glycine–HCl buffer (pH 2.0) was used as the regeneration buffer. All steps were performed according to the manufacturer’s instructions. BIA evaluation version 3.0 software (Biacore AB Corporation, Uppsala, Sweden) was used to determine the kinetic curves and calculate kinetic parameters.

### H9C2 Cell Culture

H9C2 cells, a cardiomyoblast cell line derived from embryonic rat heart tissue, were cultured in Dulbecco’s modified Eagle’s medium (DMEM, Gibco, Grand Island, NY, United States) supplemented with 10% fetal bovine serum (FBS) and 1% penicillin and streptomycin under an atmosphere of 5% CO_2_ and 95% air at 37°C. The first 20 passages of cells were used for all the experiments.

### Cell Viability Assay

Cell viability was measured using the CellTiter 96^®^ Aqueous One Solution Cell Proliferation Assay (MTS) according to the manufacturer’s protocol. As previously described (Wu et al., 2018), H9C2 cells in the logarithmic growth phase were harvested and seeded in 96-well plates at a density of 5 × 10^3^ cells/well. When cells adhered to the plates, cells were incubated for 24 h with different concentrations of Tan I (0.625, 1.25, and 2.5 μM) and then treated with 200 μM tert-butyl hydroperoxide (TBHP) for 1 h. The MTS solution was dissolved in DMEM at a ratio of 1:6 and then added to each well (100 µL/well). The cells were cultured for 1 h at 37°C, and the optical density (OD) of each well was measured using an automatic microplate reader (Gene Company, Hong Kong, China) at 490 nm.

### Reactive Oxygen Species Levels, Superoxide Dismutase Activity, and Malondialdehyde Analysis

H9C2 cells were incubated with different concentrations of Tan I (0.625, 1.25, and 2.5 µM) for 24 h and then treated with TBHP (200 μM) for 1 h. Respective commercial assay kits were used to detect levels of ROS, SOD, and MDA, according to the manufacturers’ protocols. The results of ROS were detected by using the Cell Imaging Multi-Mode Microplate Reader (Cytation5, BioTek, Vermont, United States), while the absorbance of SOD and MDA was spectrophotometrically measured at 450 and 532 nm, respectively, with an automatic microplate reader (Gene Company, Hong Kong, China).

### Apoptosis Analysis

The Annexin V-FITC Apoptosis Assay Kit was used to measure levels of H9C2 cell apoptosis according to the manufacturer’s protocol. In brief, after H9C2 cells were treated with different concentrations of Tan I (0.625, 1.25, and 2.5 μM) for 24 h and then treated with TBHP (200 μM) for 1 h, and cells were digested with trypsin without EDTA and centrifuged at 2,000 rpm for 5 min at room temperature. The collected cells were washed with pre-cooled PBS and centrifuged at 2,000 rpm for 5 min at room temperature. Next, the supernatant was discarded, and 300 μL 1 × binding buffer was added to resuspend the cells (1–5 × 10^5^ cells). Subsequently, 5 μL Annexin V-FITC staining solution were added. Following gentle mixing, the stained cells were reacted for 15 min in the dark at room temperature. Following this, 5 μL propidium iodide staining solution and 200 μL 1 × binding buffer were added and mixed well, and the samples were placed on ice. Each sample was analyzed using flow cytometry (CytoFLEX, Beckman Coulter, California, United States) within 1 h.

### Immunofluorescence Analysis

In brief, cell climbing slices of H9C2 cells were washed with PBS 3 times. The slices were fixed with 4% paraformaldehyde (PFA) for 15 min, washed with PBS 3 times, and then permeated with 0.5% Triton X-100 for 20 min at room temperature, followed by washing with PBS three times. Following this, goat serum was dripped onto the slices and blocked at room temperature for 30 min. A primary antibody was added to each slice, and the slices were incubated in a humid box overnight at 4°C, followed by washing with PBST three times. A fluorescent secondary antibody was then added to each slice, and the slices were incubated in a humid box at room temperature for 1 h and then washed with PBST three times. Finally, the nuclei were counterstained with DAPI, and the slices were incubated for 5 min in the dark, washed with PBST four times, and then mounted with mounting solution containing an anti-fluorescence quencher (Beyotime Biotechnology, Shanghai, China). A confocal microscope (LSM 880 with Airyscan, Carl Zeiss, Jena, Germany) was used to observe the cells.

### Western Blot Analysis

H9C2 cells were treated with different concentrations of Tan I (0.625, 1.25, and 2.5 µM) for 24 h and then treated with TBHP (200 μM) for 1 h. The culture medium was discarded, and the cells were washed twice with cold PBS. An appropriate amount of cell lysis solution (protease inhibitor: phosphatase inhibitor: RIPA cell lysate = 1:1:100) was then added to fully lyse the cells, which were collected in 1.5-ml EP tubes, and centrifuged at 12,000 rpm for 15 min at 4°C to obtain the protein samples. Target proteins were separated using 10% SDS-PAGE and transferred to polyvinylidene fluoride (PVDF) membranes. Tris-buffered saline containing 5% bovine serum albumin (BSA) was used to block the PVDF membranes for 1 h at room temperature. Next, the PVDF membranes were incubated with corresponding primary antibodies overnight at 4°C (Nrf2, 1:1,000; *p*-ERK1/2, 1:1,000; p-SAPK/JNK, 1:1,000; p-P38 MAPK, 1:1,000; BAX, 1:1,000; Bcl-2, 1:1,000; cleaved caspase3, 1:1,000; caspase3, 1:1,000; and GAPDH, 1:1,000) and then incubated with horseradish peroxidase-goat anti-rabbit IgG for 2 h at 4°C. GAPDH was used as an internal control, and ImageJ software (National Institutes of Health, Bethesda, Maryland, United States) was used for Western blot analysis.

### Animals and Experimental Model

Male C57 mice (6–8 weeks; 18–20 g) were purchased from the Experimental Animal Center of Southern Medical University (Guangzhou, China) and Nrf2 knockout mice housed by our research group. Animals were housed under 12-h light–dark cycle conditions at 22–26°C with free access to water and food. Animals were acclimatized for 1 week prior to experimentation. As previously described (Ning et al., 2017), mice received intraperitoneal injections of isoproterenol (ISO, dissolved in 0.9% physiologic saline, 30 mg/kg body weight) to establish an oxidative stress–induced myocardial damage model, and then, the mice were randomly divided into two groups: a model group and Tan I-dose (10 mg/kg body weight, dissolved in double-distilled water containing 2% Tween 80) group, eight per group for one week. The control group was given the same volume of double-distilled water by gavage. All operations were carried out in strict accordance with the guidelines of the Animal Care and Use Committee of Southern Medical University.

### Pathological Analysis

After one week of treatment, mice were anesthetized with sodium pentobarbital (10 mg/kg), and their hearts were excised, followed by fixation in 4% PFA. Following this, 5-μm-thick paraffin sections were prepared and subjected to hematoxylin–eosin (HE) staining and Masson’s trichrome staining, according to standard experimental protocols.

### TUNEL Apoptosis Assay

The TUNEL Apoptosis Assay Kit was used to analyze ISO-induced cardiomyocyte apoptosis according to the manufacturer’s protocol. In brief, positive staining of the nuclear area indicated apoptotic cells. Slides were analyzed using a Nikon Eclipse 80 microscope (Nikon, Tokyo, Japan) at × 400 magnification, with six fields of view randomly selected for analysis.

### Immunohistochemistry Analysis

Heart tissue sections were deparaffinized with dimethylbenzene, dehydrated using an alcohol gradient, and incubated with 3% H_2_O_2_ to block endogenous peroxidase and nonspecific binding sites. Afterward, sections were incubated with corresponding primary antibodies overnight at 4°C (Nrf2, 1:50; BAX, 1:50; Bcl-2, 1:50; caspase3, 1:50; p-ROS, 1:50). Heart tissues were observed under a microscope (400 × magnification, Eclipse E100 + Eclipse FN1, Nikon, Tokyo, Japan) using six randomly selected fields, followed by analysis using Image-Pro Plus image analysis software (Media Cybernetics, Inc. Rockville, MD, United States).

### Transthoracic Echocardiography

After one week of treatment, mice were anesthetized with 3% isoflurane. Transthoracic echocardiography was performed using a Philips iE33 Ultrasound System with a 15 MHz transducer (Bothell, WA, United States) to evaluate the shape and function of the left ventricle, including the left ventricular end-diastolic dimension (LVEDD), left ventricular end-systolic dimension (LVESD), left ventricular ejection fraction (LVEF), and left ventricular fractional shortening (LVFS). All operations were performed by one person under the guidance of a specialist.

### Data Analysis

All data are expressed as means ± S.D. and were analyzed using SPSS Statistics v. 13.0 software (SPSS Inc. Chicago, IL, United States). One-way analysis of variance (ANOVA) was used to compare measurement data among groups. Mean values were compared using an F test when variance was homogeneous. Differences between two groups were compared using the Bonferroni test. Welch’s test and Dunnett’s T3 analysis were adopted when variance was nonhomogeneous. Values of *p* < 0.05 were considered statistically significant. Each experiment was repeated three or more times.

## Results

### Tan I Inhibits Oxidative Stress and Oxidative Stress–Induced H9C2 Cell Apoptosis

**FIGURE 1:**
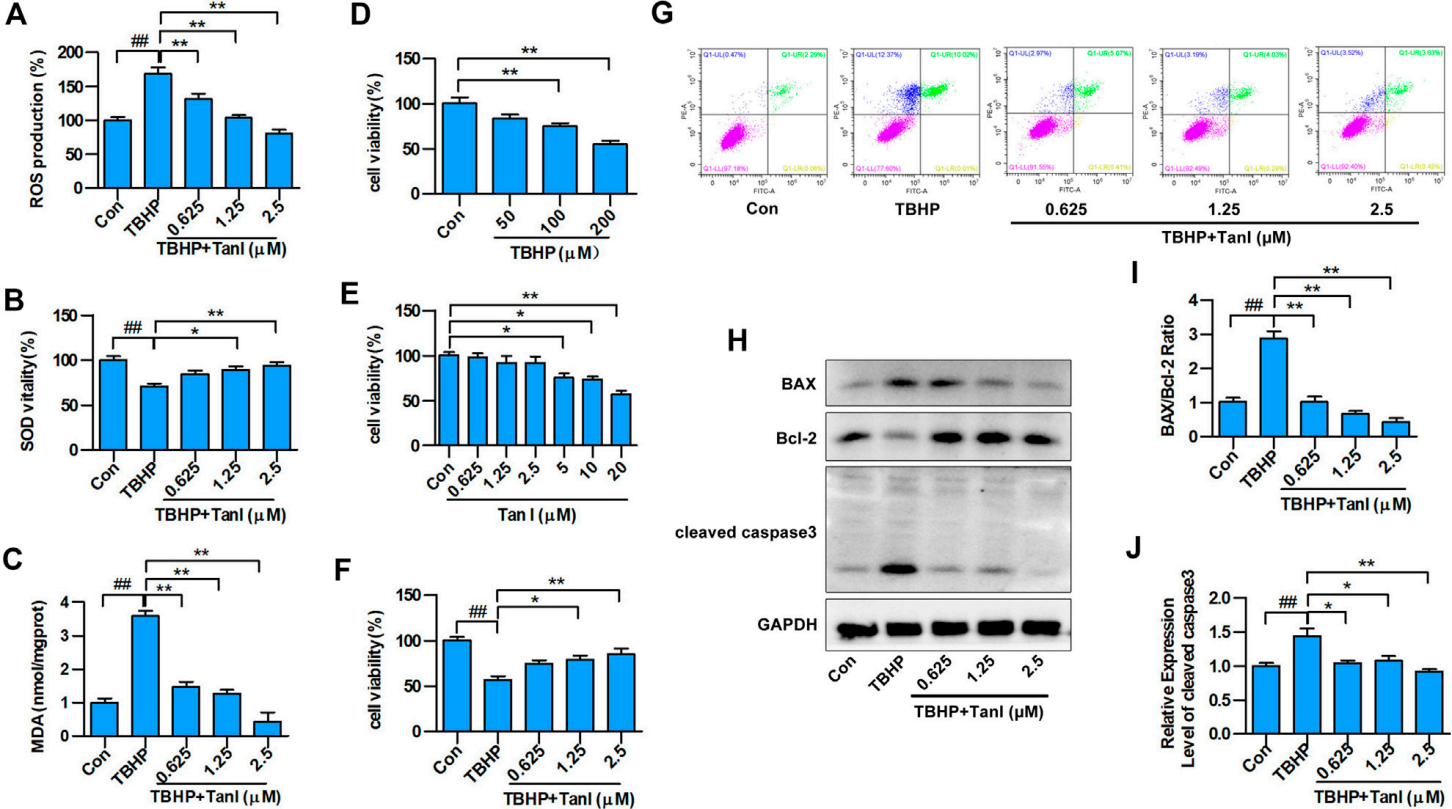
Tan I inhibits oxidative stress and oxidative stress–induced H9C2 cell apoptosis. H9C2 cells were incubated with different concentrations of Tan I (0.625, 1.25, and 2.5 µM) and then treated with TBHP (200 µM). **(A)** Reactive oxygen species (ROS) levels (*n* = 3). **(B)** Superoxide dismutase (SOD) activity (*n* = 3). **(C)** Malondialdehyde (MDA) content (*n* = 3). **(D)** Viability of H9C2 cells incubated with different concentrations of TBHP (*n* = 3). **(E)** Viability of H9C2 cells incubated with different concentrations of Tan I (*n* = 3). **(F)** Viability of H9C2 cells incubated with different concentrations of Tan I (0.625, 1.25, and 2.5 µM), followed by treatment with TBHP (200 µM) (*n* = 3). **(G)** H9C2 cell apoptosis analysis (*n* = 3). **(H)** Expression levels of BAX, Bcl-2, and cleaved caspase3 measured using Western blot analysis (*n* = 3). **(I)** BAX/Bcl-2 ratio (*n* = 3). **(J)** Relative expression levels of cleaved caspase3 (*n* = 3). Data are presented as mean ± S.D. ^*#*^ *p* < 0.05, ^*##*^ *p* < 0.01, compared to the control group. *p < 0.05, **p < 0.01, compared to the TBHP group.

To determine whether Tan I protected H9C2 cells against oxidative stress, we examined total ROS production in TBHP-treated cells. As shown in Figure 1A, ROS levels increased significantly after treatment of H9C2 cells with TBHP compared with the control group (*p* < 0.05). However, co-treatment with Tan I demonstrated significantly reduced ROS levels in a dose-dependent manner, compared with the TBHP group (*p* < 0.05). We further measured levels of oxidative stress–related biochemical enzymes, including antioxidant enzymes such as SOD, and lipid peroxidation products such as MDA. As shown in Figures 1B,C, SOD activity was significantly decreased, while MDA production was significantly increased after treatment with TBHP, compared with the control group (*p* < 0.05). However, Tan I pretreatment remarkably decreased MDA production and increased SOD activity, compared with the TBHP group (*p* < 0.05). These results confirmed that Tan I strongly affected antioxidant capacity *in vitro*.

Next, we measured the protective effect of Tan I against oxidative stress–induced H9C2 apoptosis. As shown in Figures 1D–F, H9C2 cell viability was significantly inhibited by TBHP stimulation (200 μM), compared with the control group (*p* < 0.05), but was dose-dependently enhanced by Tan I (0.625, 1.25, and 2.5 μM) treatment (*p* < 0.05). In addition, we performed flow cytometry to detect the inhibitory effect of Tan I on oxidative stress–induced H9C2 cell apoptosis. As shown in Figure 1G, cell apoptosis was significantly increased by TBHP stimulation, but dose-dependently inhibited by Tan I treatment (*p* < 0.05). We further detected expression of apoptosis-related proteins, including BAX, Bcl-2, and cleaved caspase3. As shown in Figures 1H–J, Tan I treatment significantly inhibited the expression of BAX and cleaved caspase3 and increased the expression of Bcl-2 in a dose-dependent manner (*p* < 0.05). These results indicated that Tan I suppressed oxidative stress and oxidative stress–induced H9C2 cell apoptosis.

### Tan I Directly Targets Nrf2

**FIGURE 2:**
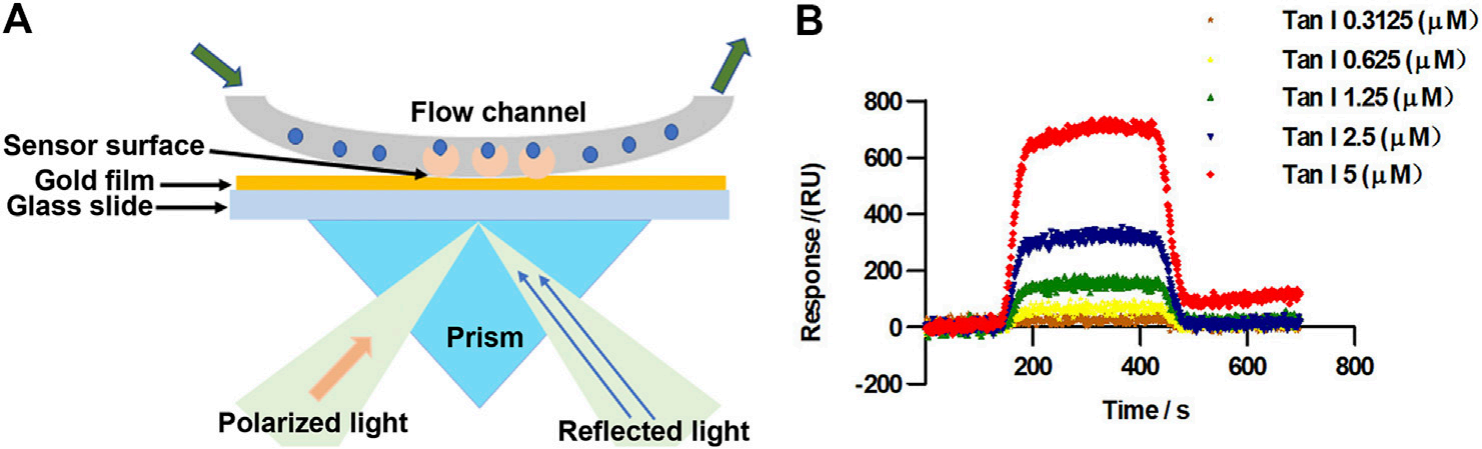
Tan I directly targets Nrf2. **(A)** The principle of surface plasmon resonance (SPR) in detection of Tan I ligand-binding affinities for Nrf2 protein. **(B)** SPR fitting curves of different concentrations of Tan I to Nrf2.

We further examined the mechanism underlying the effect of Tan I against oxidative stress and oxidative stress–induced cardiomyocyte apoptosis using a surface plasmon resonance (SPR) assay performed to explore the interaction between Tan I and Nrf2. Figure 2A represents the principle of SPR in the detection of ligand-binding affinities of Nrf2 protein toward Tan I. When polarized light from parallel surfaces is incident on the interface between two media with different refractive indices within a certain angle range, it is attenuated and totally reflected if the wave vector of the incident light is the same as that of the inner surface of the metal film. The same electronic oscillation frequency can cause resonance of free electrons in the metal. Due to the resonance, a large amount of light energy is absorbed, so that the intensity of the reflected light is significantly weakened within a certain angle. The angle at which the reflected light completely disappears within a certain angle is called the SPR angle. The SPR angle changes with the refractive index of the metal surface, and the change in the refractive index is directly proportional to the mass of the molecules bound to the metal surface. Based on the assumption that the difference in the refractive indices between different samples causes the SPR angle or SPR wavelength to change, the dynamic information of the interaction between molecules can be monitored in real time by detecting the dynamic change in the SPR angle during the reaction of biomolecules. Based on this, it is observed that Tan I directly targeted Nrf2 (Figure 2B). The binding affinity between Tan I and Nrf2 was 3.58 × 10^–6^ M, indicating that Tan I could directly bind to Nrf2. These results strongly indicated that Tan I could protect against oxidative stress and oxidative stress–induced cardiomyocyte apoptosis through Nrf2 signaling.

### MAPK Signaling Involves in the Nrf2-Mediated Oxidative Stress–Induced Cardiomyocyte Apoptosis

**FIGURE 3:**
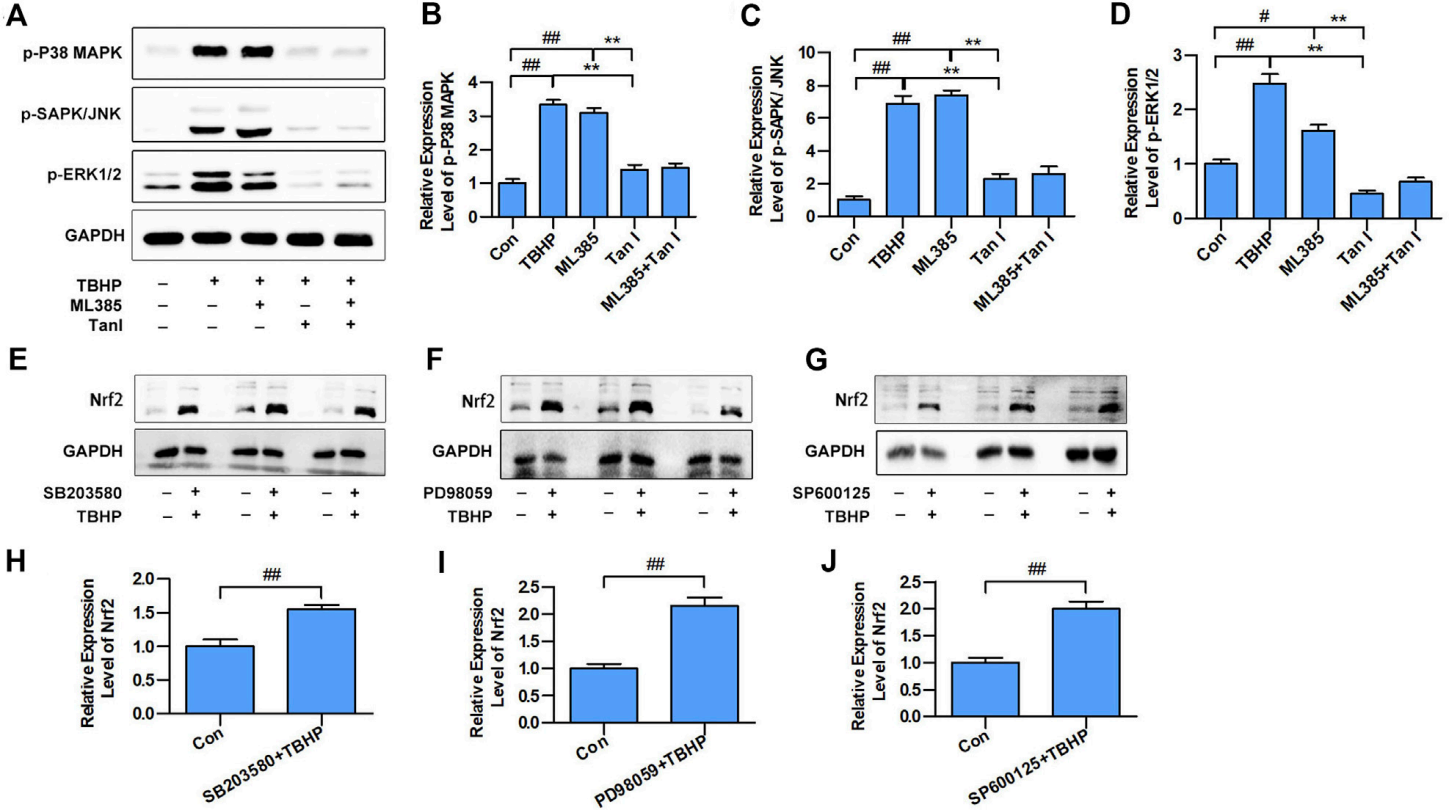
MAPK signaling involved in the Nrf2-mediated oxidative stress–induced cardiomyocyte apoptosis. **(A)** H9C2 cells incubated with Tan I (2.5 µM) and ML385 (10 µM) and then treated with TBHP (200 µM). Expression levels of p-P38 MAPK, p-SAPK/JNK, and p-ERK1/2 measured using Western blot analysis (*n* = 3). **(B)** Relative expression levels of p-P38 MAPK (*n* = 3). **(C)** Relative expression levels of p-SAPK/JNK (*n* = 3). **(D)** Relative expression levels of p-ERK1/2 (*n* = 3). **(E)** H9C2 cells incubated with SB203580 (10 µM), then treated with TBHP (200 µM). **(F)** H9C2 cells incubated with PD98059 (10 µM), then treated with TBHP (200 µM). **(G)** H9C2 cells incubated with SP600125 (10 µM), then treated with TBHP (200 µM). **(H–J)** Relative expression levels of Nrf2 (*n* = 3). Data are presented as mean ± S.D. ^*#*^ *p* < 0.05, ^*##*^ *p < 0.01*, compared to the control group. *p < 0.05, **p < 0.01, compared to the TBHP or ML385 group.

Previous studies have indicated that MAPK signaling plays a significant role in the regulation of cell apoptosis, and oxidative stress could activate MAPK signaling, thereby promoting cell apoptosis (Guo et al., 2018), which prompted us to speculate that MAPK signaling may be involved in the Nrf2-mediated apoptosis process. Three well-characterized MAPK subfamilies, P38 MAPK, SAPK/JNK, and ERK1/2, are known to be involved in cell apoptosis pathways. ML385 is a specific inhibitor of Nrf2. As shown in Figures 3A–D, inhibition of Nrf2 activated MAPK signaling, including p-P38 MAPK, p-SAPK/JNK, and p-ERK1/2. Further, SB203580, PD98059, and SP600125, the specific respective inhibitors of P38 MAPK, ERK1/2, and SAPK/JNK, activated Nrf2 (Figures 3E–J). These results suggested that MAPK signaling involved in the Nrf2-mediated oxidative stress–induced cardiomyocyte apoptosis process.

### Tan I Regulates Nrf2/MAPK Signaling to Resist Oxidative Stress and Oxidative Stress– Induced H9C2 Cell Apoptosis

**FIGURE 4:**
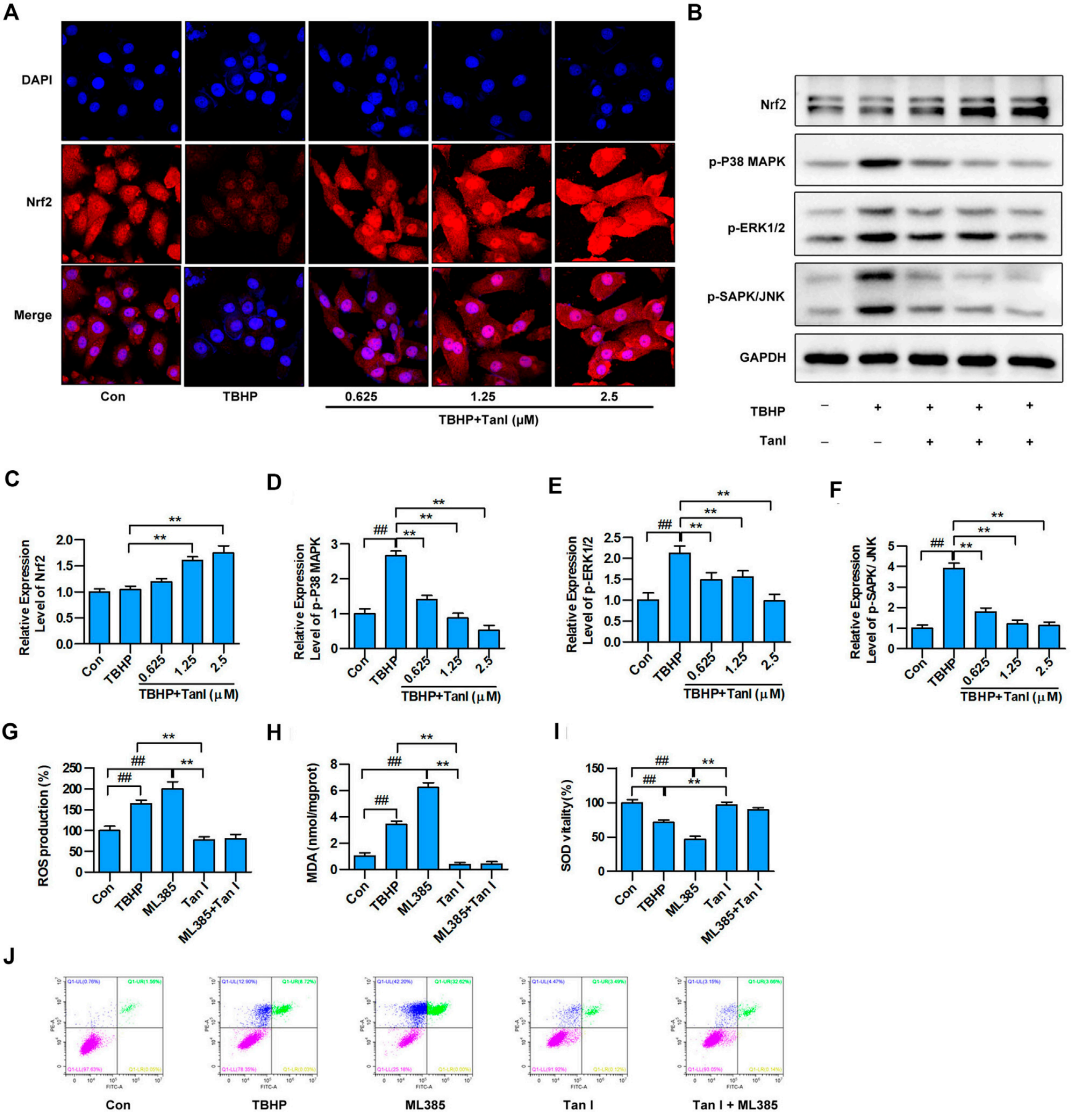
Tan I activates Nrf2 and inhibits MAPK signaling in H9C2 cells. **(A)** H9C2 cells incubated with different concentrations of Tan I (0.625, 1.25, and 2.5 µM) and then treated with TBHP (200 µM). Detection of Nrf2 transcription into the nucleus by immunofluorescence (*n* = 3). **(B)** Expression levels of Nrf2, p-P38 MAPK, p-ERK1/2, and p-SAPK/JNK, tested using Western blotting analysis. **(C)** Relative expression levels of Nrf2 (*n* = 3). (D) Relative expression levels of p-P38MAPK (*n* = 3). **(E)** Relative expression levels of p-ERK1/2 (*n* = 3). **(F)** Relative expression levels of p-SAPK/JNK (*n* = 3). **(G)** Reactive oxygen species (ROS) levels (*n* = 3). **(H)** Malondialdehyde (MDA) content (*n* = 3). **(I)** Superoxide dismutase (SOD) activity (*n* = 3). **(J)** H9C2 cell apoptosis analysis (*n* = 3). Data are presented as mean ± S.D. ^*#*^ *p* < 0.05, ^*##*^ *p < 0.01*, compared to the control group. *p < 0.05, **p < 0.01, compared to the TBHP or ML385 group.

To determine whether Tan I protected H9C2 cells against oxidative stress and oxidative stress–induced cell apoptosis through Nrf2/MAPK signaling, immunofluorescence analysis was performed to detect whether Tan I activated Nrf2. As shown in Figure 4A, Tan I treatment promoted transcription of Nrf2 into the nucleus, indicating that Tan I could activate Nrf2 and play an important role in antioxidative stress and oxidative stress–induced apoptosis. Besides, Tan I treatment significantly increased expression of Nrf2 in a dose-dependent manner, compared with the TBHP group (*p* < 0.05) (Figures 4B,C). We further measured expression levels of MAPK signaling and found that phosphorylation of P38 MAPK, ERK1/2, and SAPK/JNK increased significantly with TBHP stimulation but was dose-dependently inhibited by Tan I treatment (*p* < 0.05) (Figures 4B,D–F). These results indicated that the protective effect of Tan I against oxidative stress–induced H9C2 cell apoptosis is at least partially due to Nrf2/MAPK signaling. We further confirmed the protective effect of Tan I against oxidative stress and oxidative stress–induced cell apoptosis by activating Nrf2 signaling in H9C2 cells. As shown in Figures 4G–I, H9C2 cells treated with ML385, a specific Nrf2 inhibitor, exhibited significantly increased ROS and MDA levels and reduced SOD levels (*p* < 0.05). We further examined TBHP-induced H9C2 cell apoptosis and found that treatment with ML385 significantly increased apoptosis (*p* < 0.05) (Figure 4J). These results strongly indicated that Nrf2 signaling is involved in oxidative stress and oxidative stress–induced H9C2 cell apoptosis.

### Tan I Inhibits Oxidative Stress–Induced Myocardial Injury in the Mouse Model

**FIGURE 5:**
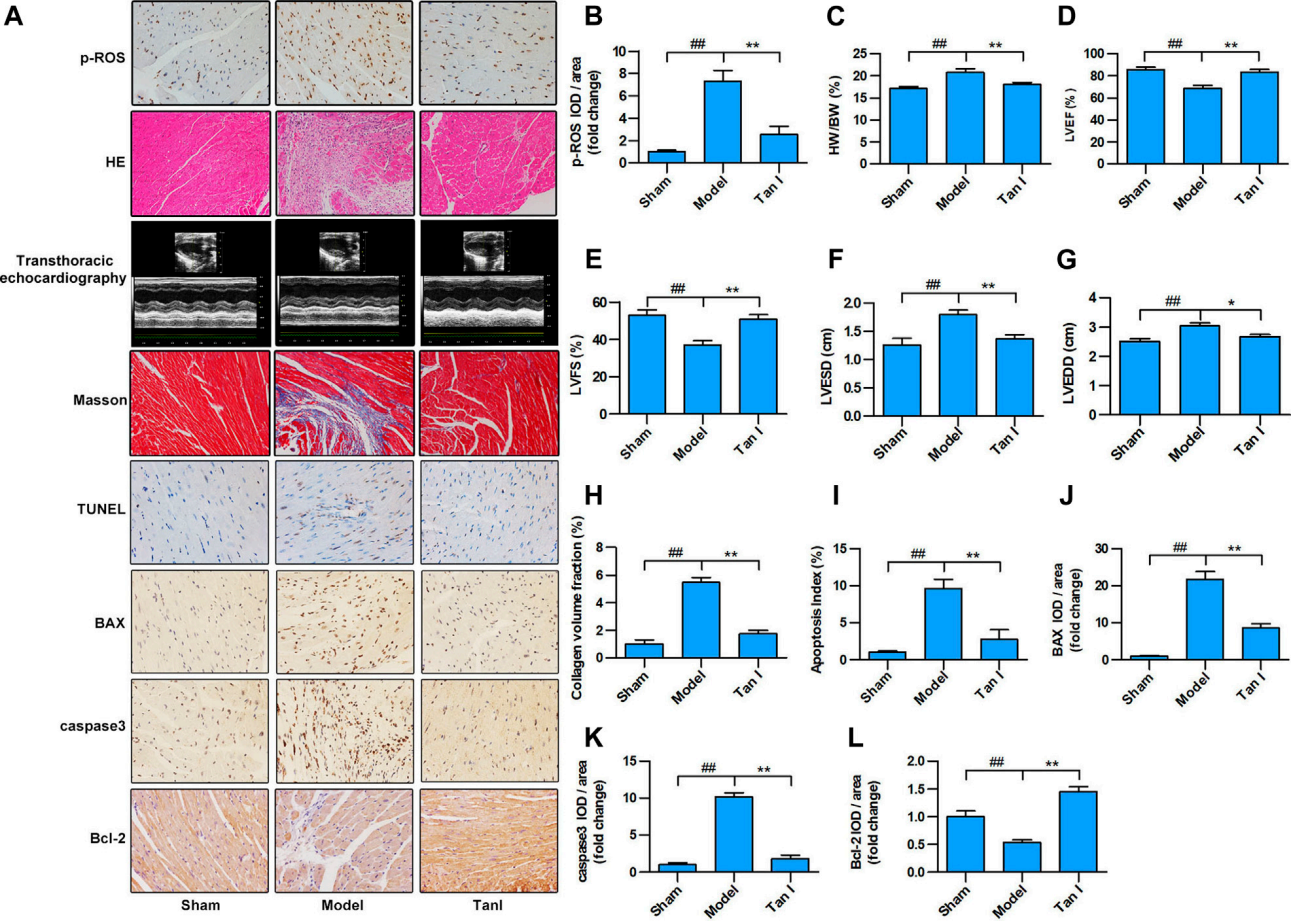
Tan I inhibits ISO-induced myocardial injury in a mouse model. Intraperitoneal injection of ISO was performed to establish an oxidative stress–induced myocardial damage model. **(A)** Hematoxylin–eosin (HE) staining; Masson’s trichrome staining; and immunohistochemistry (IHC) analysis, including p-ROS, BAX, caspase3, and Bcl-2; TUNEL analysis; and transthoracic echocardiography analysis. **(B)** Relative expression of p-ROS. **(C)** Heart weight/body weight ratio. **(D)** Left ventricular ejection fraction (LVEF). **(E)** Left ventricular fractional shortening (LVFS). **(F)** Left ventricular end-systolic dimension (LVESD). **(G)** Left ventricular end-diastolic dimension (LVEDD). **(H)** Collagen volume fraction. **(I)** Apoptotic index. **(J)** Relative expression of BAX. **(K)** Relative expression of caspase3. **(L)** Relative expression of Bcl-2. Data are presented as mean ± S.D. ^*#*^ *p* < 0.05, ^*##*^ *p < 0.01*, compared to the sham group. *p < 0.05, **p < 0.01, compared to the model group (*n* = 8).

Excessive ROS production is known to be the major cause of oxidative stress and development of CVDs. As shown in Figures 5A,B, p-ROS levels significantly increased in the ISO-induced myocardial injury model group and significantly decreased in the Tan I-dose group (*p* < 0.05). Furthermore, compared with a normal cardiomyocyte structure in the sham group, disorganized cardiomyocyte, considerable fibrous tissue hyperplasia, necrotic cardiomyocytes, and inflammatory infiltration were observed in the hearts of the model group via HE staining (Figure 5A). Interestingly, treatment of Tan I was able to significantly attenuate the ISO-induced myocardial injury mentioned above. Besides, as shown in Figure 5C, the heart weight/body weight (HW/BW) ratio significantly increased in the model group and significantly decreased after Tan I treatment (*p* < 0.05). In addition, transthoracic echocardiography revealed that LVEF (Figures 5A,D) and LVFS (Figures 5A,E) significantly decreased, but LVESD (Figures 5A,F) and LVEDD (Figures 5A,G) significantly increased in the model group compared to the sham group (*p* < 0.05). However, Tan I treatment significantly improved cardiac function compared with the model group (*p* < 0.05), which was consistent with the HW/BW ratio results. Masson's trichrome staining further revealed that collagen formation significantly increased in the model group and significantly decreased with Tan I treatment (Figures 5A,H, *p* < 0.05).

Apoptosis is a process of intrinsic cell death caused by various factors, which is attributed to programmed cell death. Many studies have shown that cardiomyocyte apoptosis plays an important role in the progression of heart failure in almost all CVDs. Improved cardiac function has been significantly associated with prevention of cardiomyocyte apoptosis. Therefore, the TUNEL apoptosis assay was performed to assess whether Tan I could suppress ISO-induced cardiomyocyte apoptosis. As shown in Figures 5A,I, few apoptotic cells were detected in the sham group, whereas significantly higher numbers were found in the model group (*p* < 0.05). However, Tan I treatment significantly decreased the number of apoptotic cells compared with the model group (*p* < 0.05). In addition, we further detected the expression of apoptosis-related proteins, including BAX, caspase3, and Bcl-2. As shown in Figures 5A,J–L, the expression levels of BAX and caspase3 were significantly increased, while the expression of Bcl-2 was significantly decreased in the model group compared with those in the sham group (*p < 0.05*); however, Tan I treatment significantly decreased the expression of BAX and caspase3 and significantly increased the expression of Bcl-2 as compared to that in the model group (*p* < 0.05), which concurred with the TUNEL apoptosis assay results. Taken together, these results strongly demonstrated that Tan I treatment could reduce oxidative stress–induced myocardial injury.

### Tan I Inhibited ISO-Induced Myocardial Injury via Regulation of Nrf2/MAPK Signaling

**FIGURE 6:**
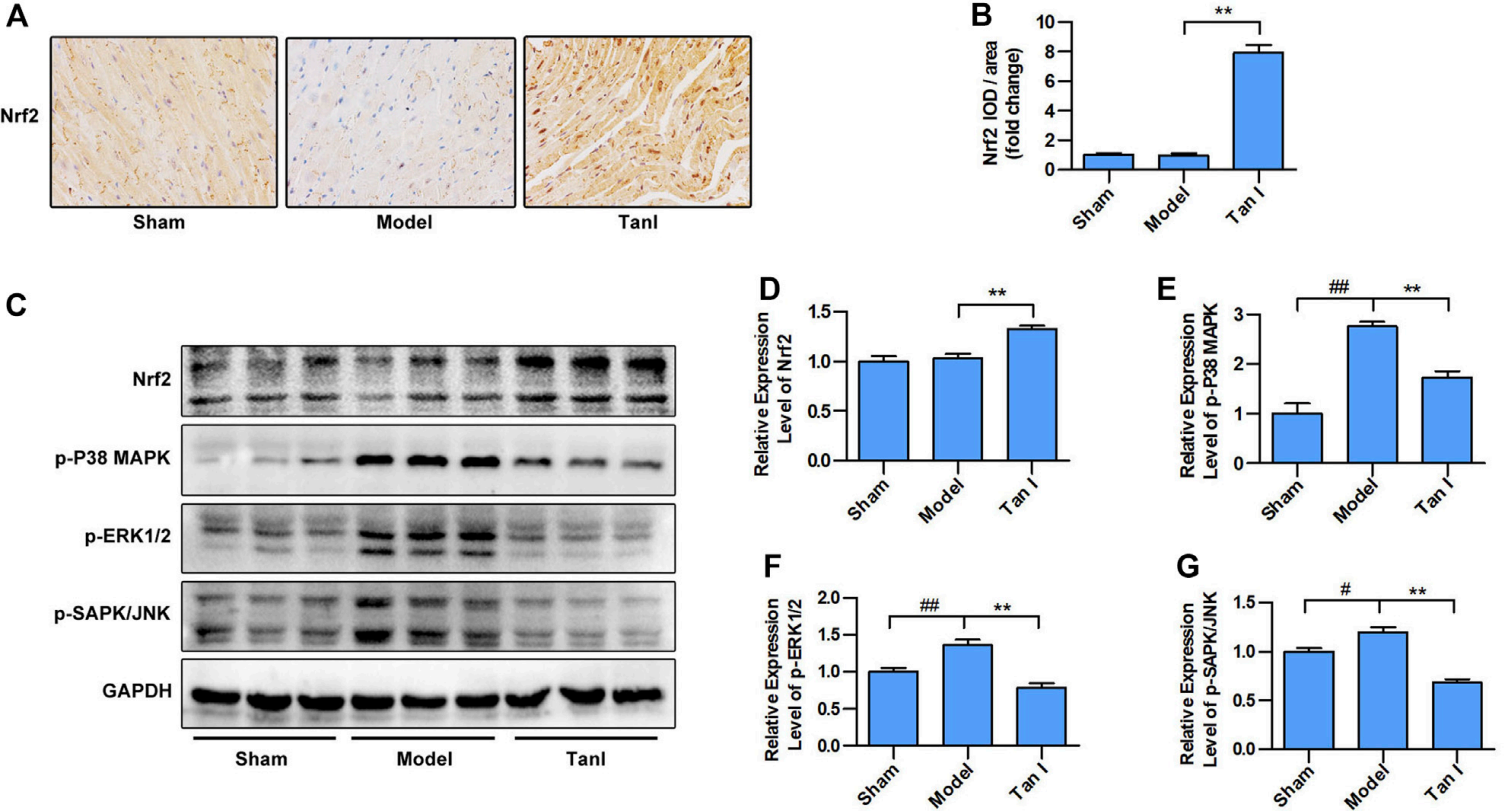
Tan I inhibits isoproterenol-induced myocardial injury by regulating Nrf2/MAPK signaling. **(A)** Immunohistochemistry (IHC) analysis. **(B)** Relative expression of Nrf2. **(C)** Expression levels of Nrf2 and MAPK signaling, including p-P38 MAPK, p-ERK1/2, and p-SAPK/JNK measured using Western blotting analysis. **(D)** Relative expression levels of Nrf2. **(E)** Relative expression levels of p-P38 MAPK. **(F)** Relative expression levels of p-ERK1/2. **(G)** Relative expression levels of p-SAPK/JNK. Data are presented as mean ± S.D. ^*#*^ *p* < 0.05, ^*##*^ *p* < 0.01, compared to the sham group. *p < 0.05, **p < 0.01, compared to the model group (*n* = 8).

We further determined whether the protective effects of Tan I against ISO-induced myocardial injury were due to regulation of Nrf2/MAPK signaling. As shown in Figures 6A,B, the results of immunohistochemistry (IHC) staining revealed that Nrf2 expression is lower in the nucleus of the model group than in the nucleus of the Tan I treatment group (*p < 0.05*), indicating that Tan I treatment could promote the nuclear translocation of Nrf2. Moreover, we found that Tan I promoted Nrf2 protein expression compared with the model group (*p* < 0.05), which was supported by the immunohistochemical staining results (Figures 6C,D). We further measured MAPK signaling expression and found that expression of p-P38 MAPK, p-ERK1/2, and p-SAPK/JNK in the model group was significantly increased compared with the sham group, while Tan I treatment significantly reduced expression of these proteins (Figures 6C,E–G, *p* < 0.05). These results indicated that Tan I inhibited ISO-induced myocardial injury by regulating Nrf2/MAPK signaling.

### Inhibition of Oxidative Stress and Oxidative Stress–Induced Cardiomyocyte Injury by Tan I is Partially Blocked in Nrf2^−/−^ Mice

**FIGURE 7:**
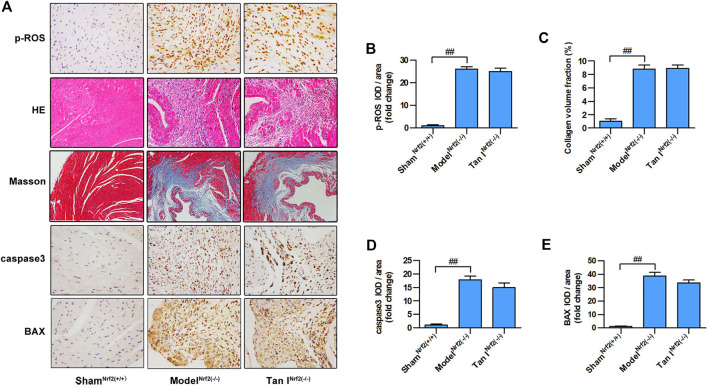
Protective effects of Tan I on oxidative stress and oxidative stress–induced cardiomyocyte injury are partially blocked in Nrf2 (^−/−^) mice. **(A)** Hematoxylin–eosin (HE) staining; immunohistochemistry (IHC) analysis including p-ROS, caspase3, and BAX; and Masson’s trichrome staining. **(B)** Relative expression of p-ROS. **(C)** Collagen volume fraction. **(D)** Relative expression of caspase3. **(E)** Relative expression of BAX. Data are presented as mean ± S.D. ^*##*^ *p* < 0.01, compared to the sham^Nrf2(+/+)^ group (*n* = 8).

Next, we used Nrf2^−/−^ mice to verify the role of Nrf2 signaling in the protective effect of Tan I against oxidative stress and oxidative stress–induced myocardial injury. As shown in Figures 7A,B, Nrf2^−/−^ cardiomyocytes were significantly more vulnerable to oxidative stress than Nrf2^+/+^ cardiomyocytes (*p* < 0.05), and the inhibitory effect of Tan I on oxidative stress was significantly blocked (*p* < 0.05). Further, HE staining revealed that ISO-induced myocardial injury was significantly aggravated in Nrf2^−/−^ mice compared with Nrf2^+/+^ mice, and the ability of Tan I to protect against oxidative stress–induced myocardial damage was significantly suppressed in Nrf2^−/−^ mice (Figure 7A). In addition, Masson’s tricolor staining revealed that collagen deposition was significantly increased in Nrf2^−/−^ mice compared with Nrf2^+/+^ mice, and the inhibitory effect of Tan I on collagen deposition was partly suppressed (Figures 7A,C, *p* < 0.05). Expression levels of BAX and caspase3 were significantly increased in Nrf2^−/−^ mice compared with Nrf2^+/+^ mice (*p* < 0.05), and the ability of Tan I to inhibit BAX and caspase3 expression was partly suppressed (Figure 7A and D-E, *p* < 0.05). These results strongly indicated that Nrf2 was the target of Tan I and that the protective effect of Tan I against oxidative stress and oxidative stress–induced myocardial injury was at least partly due to activation of Nrf2.

## Discussion

CVDs are currently the global leading cause of death, claiming more lives each year than even cancer. In the coming decades, with an aging population and increasing incidence of obesity and diabetes, the burden and medical costs associated with CVDs are anticipated to increase significantly worldwide (Benjamin et al., 2019). Although substantial efforts have been made to elucidate the pathophysiologic mechanisms governing the initiation and progression of CVDs, much work remains. As such, an improved understanding of the biomolecular mechanisms and their clinical consequences is urgently needed to reduce the burden of CVDs. Various CVDs have been shown to be associated, at least partially, with oxidative stress (Chen and Keaney, 2012). Based on the prevalence of CVDs and the role of oxidative stress in various cardiovascular pathologies, the application of naturally occurring antioxidants and the development of chemical antioxidative agents has long been of interest to ease or prevent CVDs. Unfortunately, antioxidants have shown limited therapeutic effectiveness against CVD. Therefore, the development of effective natural antioxidants remains an urgent challenge to be resolved in the current research.

Because natural products have a wide range of pharmacological effects on diseases, including CVDs, natural products are some of the most important resources used for the development of new drugs. Tanshinone, a representative natural antioxidant product, is the main compound extracted from the dried root and rhizome of *Salvia miltiorrhiza*. Along with an in-depth study of the pharmacological effects of tanshinone, abundant studies have shown that tanshinone has a variety of biological activities, including increasing the activity of antioxidant enzyme, enhancing the ability to scavenge free radicals, and reducing the production of lipid peroxides, such as MDA (Wang et al., 2020). Previous studies have shown that Tan I, one of the effective ingredients of tanshinone, exerts and inhibits oxidative stress (Jing et al., 2016). However, there are relatively few studies regarding its protective effects against CVDs. In this study, we demonstrated that Tan I exhibits the antioxidant effect *in vivo* and *in vitro*, suggesting that Tan I may be an effective natural antioxidant with a protective effect against CVDs.

Oxidative stress reflects an imbalance between ROS production and the ability of antioxidant systems to remove ROS (Panth and Paudel, 2016). Many cellular processes require low concentrations of ROS under physiological conditions; however, growing evidence indicates that chronic and acute overproduction of ROS under pathophysiologic conditions play important roles in CVD development (Valko et al., 2007; Brieger et al., 2012). Excessive ROS production causes significant damage to myocardial cells, affecting the equilibrium of the oxidant–antioxidant system, leading to apoptosis. Myocardial apoptosis during oxidative stress damage is frequently associated with excessive ROS production (Krylatov et al., 2018). Therefore, inhibiting oxidative stress–induced damage and apoptosis is an important intervention strategy for CVDs. Previous studies have demonstrated that lipid peroxidation products, such as MDA, could indirectly reflect the generation of ROS under oxidative stress, and cellular antioxidant enzymes, such as SOD, could decrease intracellular ROS content. In the present study, we found that Tan I treatment reduced MDA content and increased SOD activity, suggesting that Tan I might improve the balance of the oxidant–antioxidant system. Furthermore, we demonstrated that Tan I treatment suppressed oxidative stress–induced cardiomyocyte apoptosis. These results strongly indicated that Tan I might play a protective role against oxidative stress and oxidative stress–induced cardiomyocyte apoptosis.

Nrf2, a member of the basic leucine zipper transcription factor family, is a master transcription factor that is integral to induce endogenous antioxidant enzymes in response to oxidative stress (Done and Traustadóttir, 2016) and is widely expressed in oxygen-consuming organs, including the heart (Barančík et al., 2016). Several studies have demonstrated that loss and/or dysregulation of Nrf2 are often linked with various oxidative stress–induced diseases, including CVDs (Li et al., 2014; Strom and Chen, 2017). Under normal conditions, Nrf2 is mainly located in the cytoplasm, while it is translocated to the nucleus under conditions of oxidative stress, binds to antioxidant response elements, and then initiates activation of the endogenous antioxidative response to inhibit oxidative stress (Bellezza et al., 2018). Activation of Nrf2 signaling has been reported to increase cell antioxidative ability, which is involved in regulating cellular ROS production and scavenging (Deng et al., 2019). In the current study, we demonstrated that Tan I could activate Nrf2 to suppress oxidative stress and oxidative stress–induced cardiomyocyte apoptosis and further confirmed that inhibition or knockout Nrf2 could aggravate oxidative stress–induced cardiomyocyte damage, which concurred with previous studies. In addition, previous studies have indicated that ROS could mediate activation of MAPK signaling to promote cardiomyocyte apoptosis. In this study, we found that phosphorylation of ERK1/2, SAPK/JNK, and P38 MAPK increased significantly after TBHP stimulation in H9C2 cells. These results are consistent with those of previous research, strongly suggesting that MAPK signaling is involved in oxidative stress–induced cardiomyocyte apoptosis. Tan I treatment inhibited the activation of MAPK signaling in a dose-dependent manner. Furthermore, inhibition of Nrf2 activated MAPK signaling, including p-ERK1/2, p-SAPK/JNK, and p-P38 MAPK, while inhibition of ERK1/2, SAPK/JNK, and P38 MAPK activated Nrf2. These results suggested that Tan I could inhibit oxidative stress–induced cardiomyocyte apoptosis by activating Nrf2 and inhibiting MAPK signaling.

So far, Nrf2 could be activated through either a direct or indirect antioxidant mechanism, and most research has focused on the electrophilic indirect Nrf2 activator. Although several successful electrophilic indirect Nrf2 activators have been approved and used as the first-line oral drugs, due to the complex molecular mechanism of action of the traditional electrophilic Nrf2 activators, there may be a risk of “off-target” effects (Zhang et al., 2017). Therefore, the development of small-molecule Nrf2 agonists such as antioxidant drugs for treating various diseases remains an urgent requirement. In this study, the present findings indicated that Tan I targeted Nrf2 to protect against oxidative stress–induced cardiomyocyte apoptosis. Thus, we tested its ability to target Nrf2 directly by performing SPR analysis to assess the mechanism through which Tan I and Nrf2 interact. SPR analysis demonstrated that Nrf2 could directly bind with Tan I, which strongly indicated that Tan I directly targets Nrf2, acting as a potential Nrf2 agonist.

In summary, natural products are an important source of new drug discovery, and there are relatively few antioxidants currently used clinically to prevent and treat CVDs. In this study, we demonstrated that Tan I could protect against oxidative stress–induced cardiomyocyte apoptosis *in vivo* and *in vitro*. Mechanistic studies revealed that Nrf2 and MAPK signaling played a major role in oxidative stress–induced cardiomyocyte apoptosis, and Tan I inhibited this process, at least in part, by activating Nrf2 and inhibiting MAPK signaling activation. However, there are some limitations to our study. Previous studies have indicated that Nrf2 lies at the center of a complex regulatory network, which has been implicated in different cellular processes, such as the response to oxidative stress, apoptosis, autophagy, and ferroptosis, all of which play an important role in the occurrence and development of CVDs (Chen and Maltagliati, 2018; Dodson et al., 2019). Furthermore, natural products have the characteristic of targeting multiple molecules and complex signaling. In this study, we only studied a part of the Nrf2 functions and one of the pharmacological effects of Tan I in oxidative stress–induced cardiomyocyte apoptosis. There may be other potential mechanisms involved in its protective effects against myocardial injury. In addition, in this study, we did not investigate the safety of long-term administration of Tan I. Our future studies will be focused on conducting in-depth research on the role of Nrf2 and the pharmacological effects of Tan I both *in vivo* and *in vitro*. We hope that our study will provide a solid foundation for the clinical application of Tan I for the treatment of CVDs.

## Data Availability

The original contributions presented in the study are included in the article/Supplementary Material, and further inquiries can be directed to the corresponding authors.

## Abbreviations

BSA, bovine serum albumin; CVD, cardiovascular disease; FBS, fetal bovine serum; HE, hematoxylin–eosin; ISO, isoproterenol; LVEDD, left ventricular end-diastolic dimension; LVESD, left ventricular end-systolic dimension; LVEF, left ventricular ejection fraction; LVFS, left ventricular fractional shortening; MDA, malondialdehyde; MTS, cell viability was measured using the CellTiter 96^®^ Aqueous One Solution Cell Proliferation Assay; Nrf2, nuclear factor erythroid 2 (NFE2)–related factor 2; PFA, paraformaldehyde; PVDF, polyvinylidene fluoride; ROS, reactive oxygen species; SPR, surface plasmon resonance; SOD, superoxide dismutase; Tan I, tanshinone I; TBHP, tert-butyl hydroperoxide.
