# Supplementary material for: Tanshinone I Inhibits Oxidative Stress–Induced Cardiomyocyte Injury by Modulating Nrf2 Signaling
Source: Front Pharmacol. 2021 May 18;12:644116. doi: 10.3389/fphar.2021.644116 (PMC8167655; doi:10.3389/fphar.2021.644116)
Supplement: Supplementary file 1 [file DataSheet1.docx]

Supplementary Material

**Tanshinone I** **inhibits oxidative stress-induced cardiomyocyte injury by modulating Nrf2 signaling**

Yu-ting Wu^†^, Ling-peng Xie^†^, Yue Hua, Hong-lin Xu, Guang-hong Chen, Xin Han, Zhang-bin Tan, Hui-jie Fan, Hong-mei Chen, Jun Li

# * Correspondence: Ying-chun Zhou, Email: zhychun@126.com.

# Bin Liu, Email: xmhoolv@163.com.

## Supplementary Figures


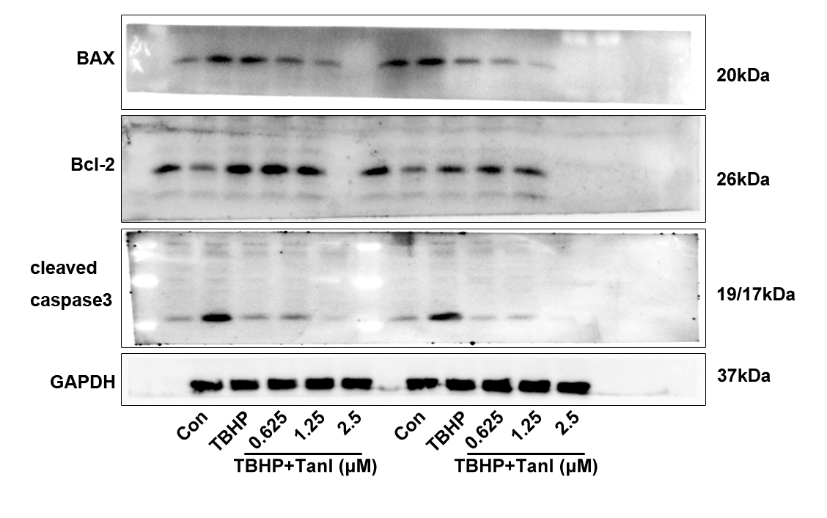


**Supplementary Figure 1.** The original pictures of western blot in Figure 1.


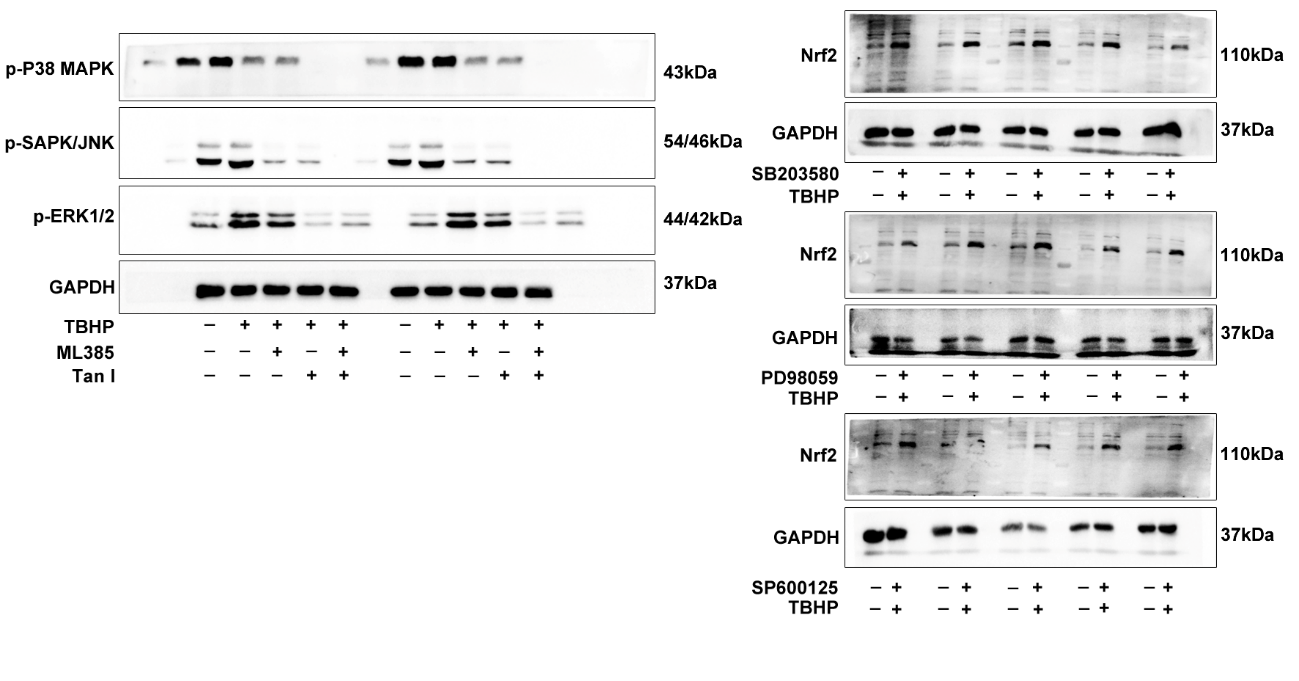


**Supplementary Figure 2.** The original pictures of western blot in Figure 3.


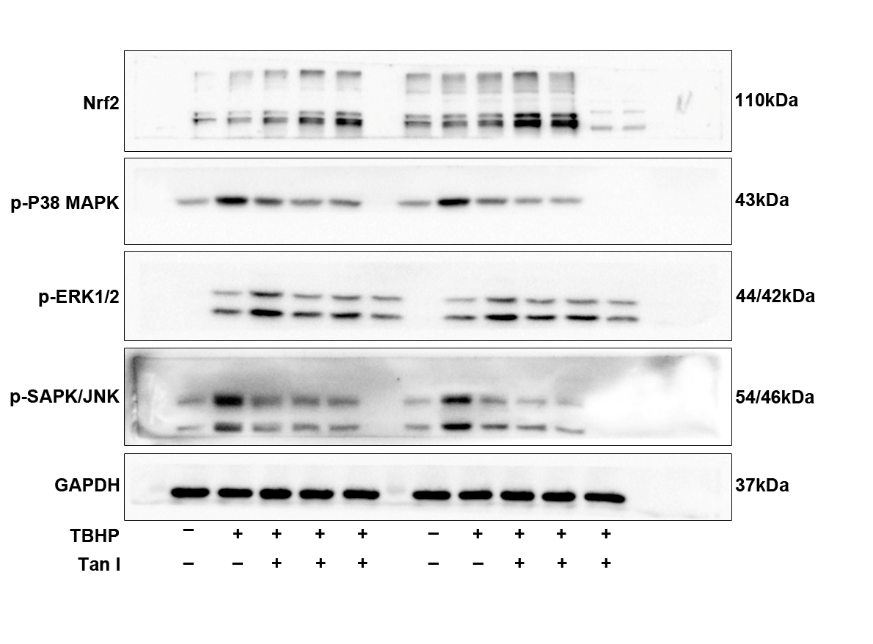


**Supplementary Figure 3.** The original pictures of western blot in Figure 4.


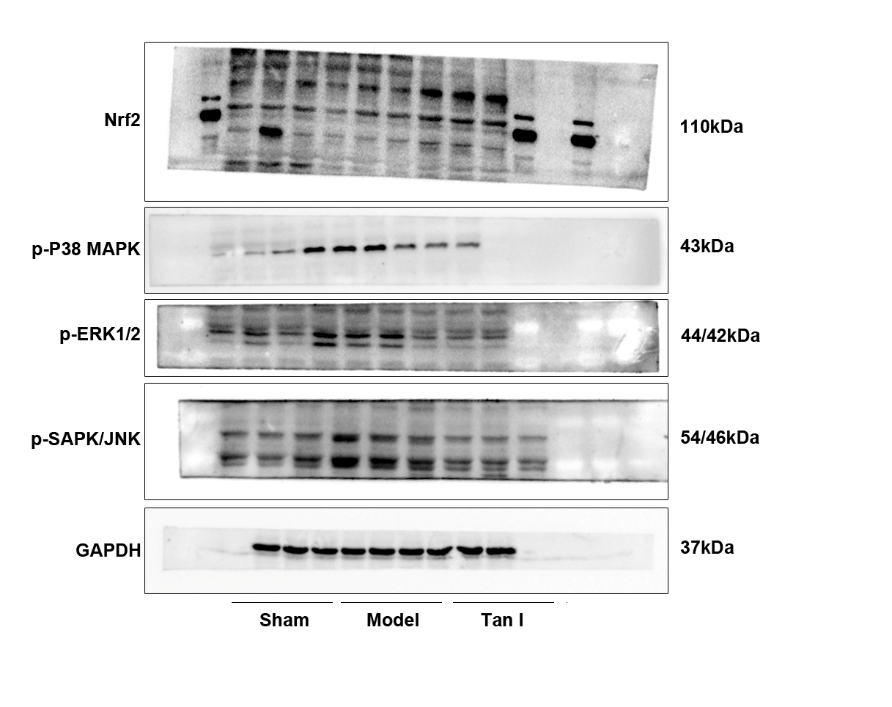


**Supplementary Figure 4.** The original pictures of western blot in Figure 6.
